# Elucidation of the Involvement of p14, a Sperm Protein during Maturation, Capacitation and Acrosome Reaction of Caprine Spermatozoa

**DOI:** 10.1371/journal.pone.0030552

**Published:** 2012-01-23

**Authors:** Pinki Nandi, Swatilekha Ghosh, Kuladip Jana, Parimal C. Sen

**Affiliations:** Division of Molecular Medicine, Bose Institute, Kolkata, India; Institut Jacques Monod, France

## Abstract

Mammalian sperm capacitation is an essential prerequisite to fertilization. Although progress is being made in understanding the physiology and biochemistry of capacitation, little has been yet explored about the potential role(s) of individual sperm cell protein during this process. Therefore elucidation of the role of different sperm proteins in the process of capacitation might be of great importance to understand the process of fertilization. The present work describes the partial characterization of a 14-kDa protein (p14) detected in goat spermatozoa using an antibody directed against the purified protein. Confocal microscopic analysis reveals that the protein is present in both the intracellular and extracellular regions of the acrosomal and postacrosomal portion of caudal sperm head. Though subcellular localization shows that p14 is mainly cytosolic, however it is also seen to be present in peripheral plasma membrane and soluble part of acrosome. Immuno-localization experiment shows change in the distribution pattern of this protein upon induction of capacitation in sperm cells. Increased immunolabeling in the anterior head region of live spermatozoa is also observed when these cells are incubated under capacitating conditions, whereas most sperm cells challenged with the calcium ionophore A23187 to acrosome react, lose their labeling almost completely. Intracellular distribution of p14 also changes significantly during acrosome reaction. Interestingly, on the other hand the antibody raised against this 14-kDa sperm protein enhances the forward motility of caprine sperm cells. Rose-Bengal staining method shows that this anti-p14 antibody also decreases the number of acrosome reacted cells if incubated with capacitated sperm cells before induction of acrosome reaction. All these results taken together clearly indicate that p14 is intimately involved and plays a critical role in the acrosomal membrane fusion event.

## Introduction

After leaving the testis, mammalian spermatozoa from many species are morphologically differentiated but have acquired neither progressive motility nor the ability to fertilize a metaphase II-arrested egg. During epididymal transit, sperm acquire the ability to move progressively; however, they are still fertilization incompetent. Fertilization capacity is gained after residing the sperm in female reproductive tract for a finite period of time. The physiological changes that confer on the sperm the ability to fertilize are collectively called capacitation [1]. Capacitation includes several cellular changes in the sperm particularly in the distribution and composition of certain glycoproteins, protein tyrosine phosphorylation, intracellular Ca**^2+^** and cAMP concentrations, as well as motility pattern [2], [3]. This phenomenon is an absolute prerequisite that spermatozoa must undergo in order to interact efficiently with the zona pellucida and to accomplish one of the last steps leading to fertilization, namely the acrosome reaction (AR) [4].

The acrosome is an exocytotic vesicle overlying the anterior region of the sperm head and contains a variety of proteins, including several protease zymogens, protease inhibitors, zona pellucida (ZP) binding proteins, and other ligand-binding proteins [5]. Only capacitated sperm cells are able to undergo the zona-triggered AR and this process characteristically involves multipoint fusions of the sperm head plasma membrane (PM) with the outer acrosome membrane (OAM) [6], [7]. This leads to the release of various hydrolytic enzymes principally, the trypsin like acrosin [2] and in the elimination of various surface antigens that are normally exposed on the acrosomal cap of spermatozoa and allows zona pellucida penetration [4]. Only acrosome reacted sperm can penetrate the ZP and fuse with egg plasma membrane [2].

Though sperm membrane modification occurs throughout the male reproductive tract, caput and corpus epididymis are involved in the acquisition of sperm fertilizing ability whereas the cauda segment are specialized in sperm storage [8]. During epididymal maturation, sperm membrane lipids undergo distinct physical and chemical alteration [9]. Changes in the distribution of sperm membrane protein occurring during this process reflect biochemical alterations of both membrane lipids and proteins. The sperm plasma membrane has both membrane integrated and surface adsorbed proteins when spermatozoa leave the testis. Some of these surface proteins change their location from one membrane domain to another during sperm maturation. Other sperm surface proteins are altered, masked or replaced by new proteins of epididymal origin [10]. Identification of epididymal sperm proteins involved in acquisition of sperm fertilizing ability has been investigated in many laboratories [11]. Previously, a low molecular weight 14 kDa protein from goat spermatozoa, named as p14, has been characterized and reported from our laboratory [12]. In the present study we have investigated the localization of p14 on caprine spermatozoa along the epididymal duct and established whether epididymal transit, capacitation or the acrosome reaction affect the distribution of this particular protein. The role of this protein, if any, in sperm motility and acrosome reaction has also been investigated.

## Materials and Methods

Sodium lactate, bovine serum albumins, calcium ionophore A23187, Triton-X-100, EDTA, EGTA, beta-mercaptoethanol, SDS, protease inhibitor cocktail, Na_3_VO_4_, glycerol, leupeptin, aprotinin, PMSF, PVDF membrane, goat-antirabbit IgG (alkaline phosphatase conjugated), and FITC-conjugated goat anti-rabbit-IgG, Rose-Bengal stain, kanamycin, penicillamine, TPCK, TLCK, DEAE-cellulose, thioglycerol, TEMED, cacodylate were obtained from Sigma Chemicals, USA. Sodium chloride, sodium carbonate, sodium bicarbonate, calcium chloride, heparin, sodium pyruvate, glucose, magnesium chloride, ATP, BCIP, NBT, DMSO, HEPES, magnesium sulphate, potassium chloride, potassium phosphate, Tris, benzamidine, CHAPS, glutaraldehyde, xylene, sucrose were purchased from Sisco Research laboratory, India. Paraformaldehyde, Tween-20, methanol, dimethylformamide, acetic acid, trichloroacetic acid, Bismarck Brown were obtained from Merck, India. Freunds complete and incomplete adjuvants, Micro BCA Protein Assay Reagent kit were purchased from Genei, Bangalore, India. All other chemicals used were of analytical grade. Deionized milli-Q water was used in all experiments.

### Ethics statement

This study was carried out in strict accordance with the recommendations in the Guide for the Care and Use of Laboratory Animals of the National Institutes of Health. All experimental animal protocols received prior approval from the Institutional Animal Ethical Committee (Bose Institute, Registration Number: 95/99/CPCSEA).

### Collection of caprine epididymis

Fresh epididymides of adult goats were obtained from the local slaughterhouse (Haji slaughter house, CK Block, Sector 1, Salt Lake, Kolkata 700064, India) immediately after sacrifice and brought to the laboratory on ice. Sacrifice of the animals was done following local municipality's guidelines. Spermatozoa were extracted from the tissue within 2 hrs of slaughtering of the animals.

### Preparation of spermatozoa and epididymal plasma

Spermatozoa were obtained from goat cauda (or other regions as and when required) epididymis using standard procedure. Highly motile spermatozoa were extracted at room temperature from the goat epididymis in a modified Ringer's solution (RPS medium: 119 mM NaCl, 5 mM KCl, 1.2 mM MgSO_4_, 10 mM glucose, 16.3 mM potassium phosphate, 50 Units/ml penicillin, pH 6.9). Number of spermatozoa in the sample was estimated with a haemocytometer. Freshly extracted sperm preparations contained about 10–20×10^7^cells/ml. For the preparation of goat cauda epididymal plasma (EP), freshly extracted sperm preparation was centrifuged at 800×g for 10 mins. The supernatant was spun again at 12000×g to obtain cell-free EP. The concentration of EP was expressed as its protein content [13].

### Induction of capacitation and acrosome reaction

Highly motile swim-up spermatozoa were extracted at room temperature from the cauda epididymis in Tyrodes medium (100 mM NaCl, 3.1 mM KCl, 1.5 mM MgCl_2_, 0.29 mM potassium dihydrogen phosphate, 21.6 mM sodium lactate,1 mM sodium pyruvate, 20 mM HEPES, 50 µg/ml Kanamycin, pH 7.4). Control samples (non capacitated, NC) were taken immediately following swim-up procedure for processing by immunoblotting and immunofluorescence.

In vitro capacitation was performed by incubating the swim-up samples (2×10^8^cells/ml) in modified Tyrodes medium (above Tyrodes medium plus 2 mM CaCl_2_, 7 mg/ml BSA and 25 mM NaHCO_3_, 10 µg/ml heparin pH 7.4) for 3 hrs at 39°C in a humidified incubator with 5% CO_2_ in air (C) [14]–[16].

Acrosome reaction was induced by the addition of 3 µM calcium ionophore, A23187 in 0.3% DMSO to the capacitated sperm (2×10^8^cells/ml) and incubated further at 39°C for 1 hr (AR samples) [17]. Control tubes were run in presence of DMSO but without any ionophore, which was found to have no effect. To study the effect of anti-p14 antibody on acrosome reaction, cells were incubated with the same for 1 hr at room temperature before induction of acrosome reaction. Acrosomal status was then assessed using Rose Bengal staining method.

### Extraction of proteins

To prepare whole-cell extract aliquots of 0.5 ml (10^8^ cells) of noncapacitated, capacitated and acrosome reacted samples were centrifuged in a microfuge at 7500×g for 5 mins at room temperature, the supernatant was discarded. The resulting sperm pellet was resuspended in 500 µl extraction medium (2% SDS, 28% sucrose, 12.4 mM N,N,N′,N′-tetramethylethylenediamine and 185 mM Tris–HCl, pH 6.8 [18] and immediately incubated for 5 mins at 100°C. After centrifugation at 7500×g for 5 mins, the supernatant was recovered. The concentration of proteins in the supernatant was measured using the Micro BCA protein assay reagent kit. To the supernatant, 2-mercaptoethanol and glycerol were added to a final concentration of 5 and 1% respectively. Finally, extracts were incubated at 100°C for 5 mins and then stored at −20°C until used for Western blot analysis [17].

The cytosol from different epididymal region was prepared following the method previously standardized in our laboratory [12]. Briefly, the tissue was homogenized in 50 mM Tris–HCl buffer (pH 7.5) containing 0.25 M sucrose, 1 mM EDTA, 1 mM EGTA, 1.5 mM thioglycerol and 0.1 mM TPCK, 0.1 mM TLCK, 1 mM PMSF, 10 µg/100 mL leupeptin as protease inhibitors. The homogenized mixture was centrifuged at 12000×*g* for 20 mins at 4°C, supernatant collected and centrifuged at 30000×*g* for 30 mins at 4°C. The post 30000× g supernatant was subjected to ultracentrifugation at 100000×*g* for 1 hr at 4°C. The post 100000×g supernatant, termed as ‘cytosol’, was collected and used for Western analysis.

Individual sperm cell was fractionated to cytosol and membrane. First centrifugation was done at 4300×g for 10 mins at 4°C. The pellet was resuspended in 20 mM Tris-HCI (pH 7.5), 0.25 M sucrose, 2 mM EGTA, 1 mM EDTA, 1 mM benzamidine, 1 mM Na_3_VO_4_, 10% glycerol, 25 µg/ml leupeptin, 4 pg/ml aprotinin, and 1 mM PMSF (homogenization buffer). Cell suspensions were then sonicated (30-sec pulse, power setting 4, Sartorius made). The homogenate was centrifuged for 10 mins at 10000×g for pelleting cell debris. The resulting supernatant was centrifuged at 100000×g (60 mins, 4°C) to separate cytosolic fraction and membrane component. The cytosolic fraction was concentrated to at least 1/10th of the original volume using a microconcentrator 30 (Amicon, USA). The membrane fraction was resuspended in the homogenization buffer supplemented with 0.6% 3-[(3-cholamidopropyl) dimethylammonio]-l-propanesulfonate (CHAPS) [19].

The plasma membranes and soluble components of the acrosome were fractionated from the sperm pellet following the described method [5], [20]. Briefly, sperm were resuspended at a concentration of 20×10^6^/ml in 0.625% Triton X-100 and 0.15 M NaCl, 5% sucrose, protease inhibitor cocktail and 20 mM sodium acetate, pH 5.2 (5 mins, 4°C). The sperm suspension was further homogenized by passing through a 26-gauge syringe needle (twice). The sperm suspension was separated into soluble and insoluble fractions by centrifugation (10 000× *g*, 10 mins, 4°C). Each fraction was subjected to protein quantification using BCA protein estimation and to SDS-PAGE/immunoblotting. Peripheral plasma membrane proteins from intact caudal epididymal sperm were extracted by treatment with a sucrose solution (320 mM) containing 1 mM ATP, 1 mM EDTA, and 0.2 mM *N*a-*p*-tosyl-L-lysine chloromethylketone hydrochloride (AES). Approximately 70×10^6^ mouse sperm were incubated in 0.5 ml of AES for 20 mins at 4°C. Sperm were then centrifuged (600× *g*, 10 mins, 4°C), and the collected AES supernatant was concentrated for immunoblotting [20].

### Raising antibody against p14

Antibody was raised against p14, purified from goat cauda epididymal cytosol, by immunizing 1-year-old white albino rabbit with the protein [21]. All experimental animal protocols received prior approval from the Institutional Animal Ethical Committee (Bose Institute, Registration Number: 95/99/CPCSEA).

In first shot, 100 mg of the protein was mixed with Freund's complete adjuvant (1∶1) and intramuscular injection was given. The subsequent three booster doses were given 10 days apart with 250 mg protein mixed with Freund's incomplete adjuvant (1∶1) each time. Fifth shot (intravenous) with pure antigen only was given one month following the fourth one.

Rabbit was bled from marginal ear vein 10 days following fourth and fifth shot and serum was collected for subsequent study [22].

The immunoglobulin of the immune serum was precipitated twice with 50% ammonium sulfate. The final precipitate was dissolved in 0.01 M PBS, pH 7.0, and excess ammonium sulfate was removed by dialysis against the same buffer. The immunoglobulin fraction obtained after the salt fractionation was subjected to DEAE-cellulose chromatography. Unbound protein peak containing IgG was eluted with 0.01 M phosphate buffer at pH 7.0. This purified anti-p14 antibody was used for subsequent immunological studies.

### Flow cytometry analysis

The extent of p14 expression in goat sperm was determined from flow cytometric analysis (BD-FACS calibur) following established procedure. Sperm were stained with anti-p14 polyclonal antibody at a final concentration of 1∶1000, and the secondary antibody, fluorescein isothiocyanate (FITC)-conjugated goat antibody against rabbit IgG (Sigma) at a dilution of 1∶50. At least 10000 individual sperm per sample were analyzed for FITC fluorescence emission.

### Western blot analysis

Western blot analysis of protein associated with spermatozoa was done following standard protocol. Briefly, equal amount of protein from extracts of the caput, corpus and cauda epididymis and spermatozoa from each section were resolved over 16.5% SDS-polyacrylamide gels, transferred onto PVDF membranes, probed with rabbit polyclonal antisera against p14 (1∶1000), the bound IgG was detected with goat anti-rabbit-ALP (Sigma), immunoreactive band was visualized using NBT-BCIP as a chromogenic substrate for alkaline phosphatase. Similar procedure was followed in case of NC, C and AR samples.

### Confocal Microscopy study

Immunohistochemistry was carried out for p14 localization and expression in different parts of the epididymis. Fixed epididymis samples were dehydrated in gradient ethanol, treated with xylene, and finally embedded in paraffin wax. Serial paraffin sections (5 µm) were deparaffinized in xylene and rehydrated in decreasing concentrations of ethanol. To prevent background staining, sections were incubated in normal goat serum at room temperature for 20 mins after being washed in phosphate buffer saline (PBS, 0.01 M pH 7.2). Following fixation and permeabilization, incubation was continued for overnight at 4°C with polyclonal rabbit anti-p14 as primary antibody (1∶1000 dilution) in PBS buffer (0.01 M, pH 7.2). After washing in PBS buffer, sections were treated with the green-fluorescein isothiocyanate (FITC) conjugated goat-anti-rabbit IgG which was previously incubated with primary antibody. Negative controls were run in parallel under identical conditions, but either in the absence of primary antibody or in the presence of normal rabbit serum. Finally, the sections were imaged using a Zeiss Confocal Microscope (Carl Zeiss, Germany).

### Indirect immunofluorescence to localize p14 on noncapacitated, capacitated and acrosome reacted sperm cells

The localization of p14 was determined by immunofluorescence with washed spermatozoa or cells incubated in capacitation/acrosome reaction condition. In the first set of experiments, the spermatozoa were washed with PBS (pH 7.2) and fixed for 30 mins in 3.7% paraformaldehyde. After washing the cells with PBS, cells were permeabilized with 0.25% Triton-X-100 for 15 mins, washed thrice with PBS. Non-specific binding sites were blocked with 3% BSA in PBS for 1 hr at 37°C in a humid chamber. After blocking, cells were washed three times with PBS and incubated with either purified anti-p14 antibody or pre-immune sera (negative control) for 2 hrs at 37°C. Following extensive washing in PBS, the cells were incubated for 2 hrs at room temperature with fluorescein isothiocyanate (FITC)-conjugated secondary antibody raised in goat. Next, cells were washed thrice with PBS; smear prepared on a glass slide with a drop of cell suspension and allowed to dry. 10 µl antifade mounting media (invitrogen) was added on the dried smear, covered with a coverslip and sealed by nail polish. Spermatozoa were examined by confocal microscopy (Zeiss laser scanning confocal microscope, LSM-510). Noncapacitated swim-up cells (NC), capacitated cells (C) and acrosome reacted cells (AR) were processed similarly to localize p14 there.

In another set of experiments, the presence of p14 was investigated in nonpermeabilized cells to measure the surface expression of the protein. After two washings, a 100-µl aliquot was incubated with the antibodies as described above for 2 hrs at room temperature. The cells were next washed twice by centrifugation (250×*g*, 10 mins), resuspended in PBS. The FITC-conjugated secondary antibody was added to the suspension, and the spermatozoa were incubated for 2 hrs. Finally the spermatozoa were washed twice in PBS by centrifugation (250×*g*, 10 mins), and mounted on slides for analysis as described above. Noncapacitated swim-up cells, capacitated cells and acrosome reacted cells were processed similarly.

### Microscopic analysis of sperm motility

Spermatozoa showing forward progression were identified under a phase contrast microscope at 400× magnification. An aliquot of the freshly extracted sperm preparation (1×10^6^ cells) was incubated with anti-p14 antibody in a total volume of 0.5 ml RPS medium for 15 mins at room temperature before assessing the sperm motility with a haemocytometer. For the microscopic method of assay of sperm motility (expressed as %), all cells which showed some degree of motility (vibrating, progressive motility) was counted. Experiments were repeated four times.

### Preparation of sperm sample for assay in CASA

The assay conditions were same as that of forward motility assay under phase contrast microscope. Total cell numbers were counted under a phase contrast microscope at 400× magnification in a hemocytometer. Spermatozoa (0.5×10^6^cells) were incubated in the absence or presence of appropriate dilution of anti-p14 antibody at 32°C±1 temperature for 15 mins in a total volume of 0.5 ml of Ca**^2+^** free modified Ringer phosphate solution.

### Sperm motility study following CASA

After preparation of sperm sample, analysis was performed using the CASA system (Version: 12, HTM-CEROS CASA System, Hamilton Thorne Research, Inc., Beverly, MA, USA) for assessing the sperm motility following incubation with anti-p14 antibody. Briefly, a 5 µl aliquot of prepared sperm sample was placed on a Mackler chamber. At least 200 spermatozoa were counted with CASA to evaluate the sperm motility variables including Average Path Velocity (VAP), Straight Line Velocity (VSL) and Curvilinear Velocity (VCL). The CASA settings were followed according to the manufacturer's instruction.

### Rose Bengal Staining

The conventional “acrosome reaction” is based on the detection of the acrosomal glycoproteins following interaction with Rose Bengal dye [23] with following modification [24]. Following termination of acrosome reaction with 3% glutaraldehyde, the cell suspension was incubated at room temperature for 2 hrs. Samples were centrifuged at 800×g for 3 mins and the supernatant was aspirated. The pellet was resuspended in PBS and washed twice with the same medium. Smear was prepared with a drop of suspension and air-dried. The slides were stained with 0.8% Bismarck brown in deionized water (pH 1.8 with 2N HCl) at 37°C for 25 mins and rinsed with distilled deionized water. Finally the slides were stained for 25 mins in 0.8% Rose Bengal in 0.1 M cacodylate buffer, pH 6.0 for detection of the glycoprotein content of the intact acrosomal sac.

The slides were then washed with deionized water, dehydrated in increasing concentration of alcohol (%), cleared in xylene and mounted with paramount and cover slip. A total of 200–500 spermatozoa were evaluated and recorded as either “acrosome reacted” (un-intact) sperm (no colored spot on tip of sperm head) or “acrosome un-reacted” (intact) sperm (with a colored spot on tip of sperm head). The experiments were repeated four times.

### Statistical analysis

Values are shown as standard error of mean (SEM) except otherwise indicated. Data were analyzed and, when appropriate, significance of the differences between mean values was determined by a Student's t test. Results were considered significant at p<0.05.

## Results

### Presence of p14 in caprine sperm cells and in epididymal epithelial tissue

**Figure 1:**
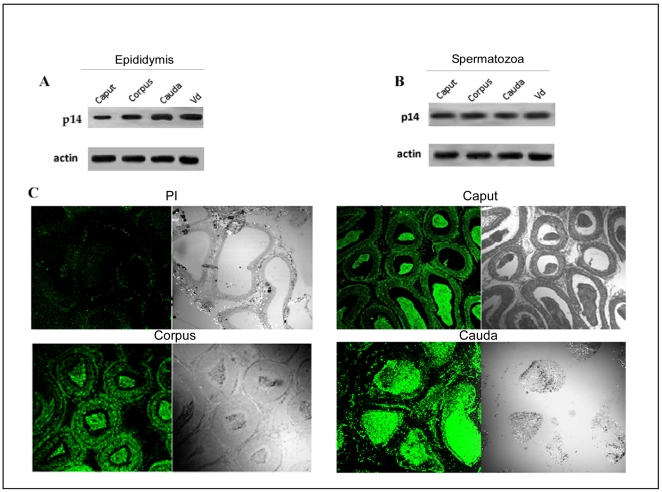
Presence of p14 in caprine sperm cells and in epididymal epithelial tissue. Rabbit polyclonal antisera were raised against p14 protein (antigen), purified by affinity purification procedure and was used for Western blotting. (**A**) Western blot analysis of p14 in total tissue extracts from caput (Cap), corpus (Cor) and cauda (Cau) of the epididymis and vas deferens (Vd). Blot was probed with monoclonal antibody against actin to assess protein loading. (**B**) Western blot analysis showing p14 in total protein extracts of sperm from caput (Cap), corpus (Cor) and cauda (Cau) regions of the epididymis and vas deferens (Vd). Actin was used as loading control. (**C**) The immunohistochemical staining to show the expression pattern of p14 in the epithelial cells and lumen of the caput, corpus and cauda epididymis. Preimmune serum at the same condition showed no immunoreactivity. All the analysis was performed four times.

p14 was previously purified and biochemically characterized from goat cauda epididymis [12]. With this background knowledge, we have examined the presence of p14 by Western blot analysis in caprine epididymis and epididymal spermatozoa using a polyclonal anti-p14 antibody raised in rabbit in our laboratory. The sensitivity and specificity of the antibody were previously verified by Western blot analysis. As evident from Figure 1A, p14 expression was distinctly observed and was found to gradually increase in the protein extract obtained from the caput, corpus and cauda epidydimis region as well as in the vd. Similar expression pattern was also observed in the sperm protein extracts obtained from the caput, corpus, cauda and vd regions though the change in the expression level was not very prominent (Figure 1B).

### Localization of p14 in epididymal epithelial tissue and spermatozoa

**Figure 2:**
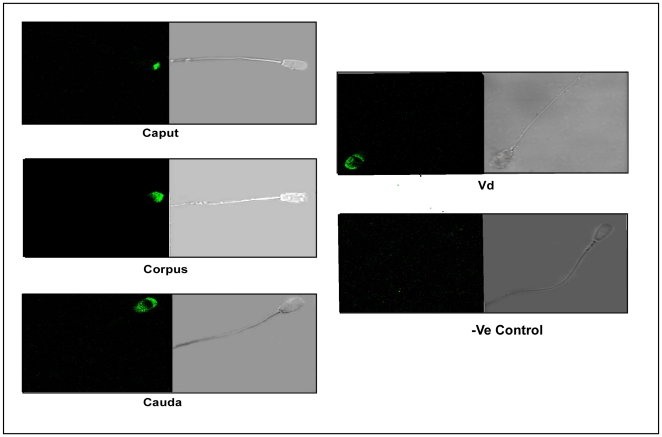
The localization of p14 on sperm surface. The localization of p14 on the surface of spermatozoa by indirect immunofluorescence analysis. The subcellular localization of p14 was determined using anti-p14 antibody and immunofluorenscence of p14 (FITC-labeled, green) was shown by confocal microscopy. The positive p14 immunoreactivity was localized to the anterior-acrosomal region of live caput and corpus sperm whereas in cauda and vd spermatozoa, it is localized on anterior as well as post acrosomal region of sperm head. Experiments were repeated four times.

**Figure 3:**
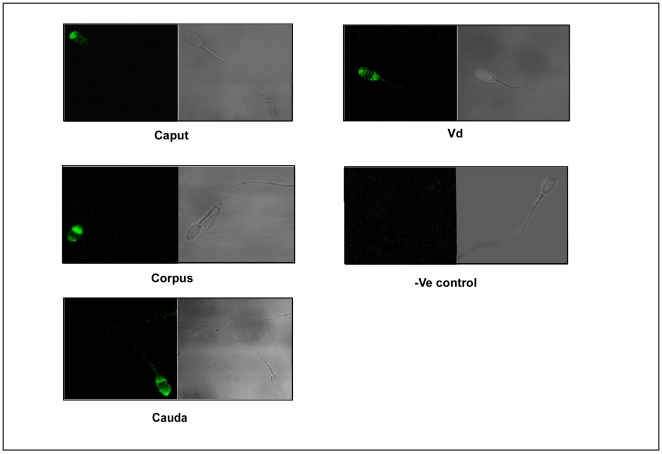
The localization of p14 protein in intracellular region of caprine spermatozoa. The localization of p14 in intracellular region of spermatozoa by indirect immunofluorescence analysis. The localization of p14 was studied as in Figure 2 except that fixed/permeabilized cells were used for analysis. Experiments were repeated four times.

**Figure 4:**
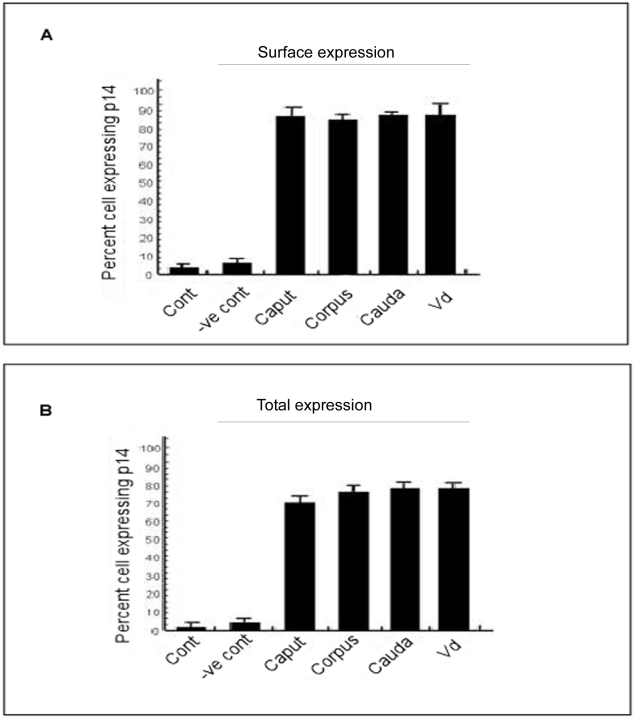
Flow cytometric analysis indicating the presence of p14 in sperm population. FACS analysis showing the percent expression of p14 in caput, corpus, cauda and vd spermatozoa under live and fixed-permeabilized condition. Details are described in the text. Experiments were repeated four times.

The western blot analysis was further verified using immunohistochemical studies.The positive signal was localized to epididymis and was present both in luminal sperm cells and epithelial cells (Figure 1C). Confocal microscopic analysis of live and fixed-permeabilized spermatozoa suggested that for live cells (Figure 2), p14 was present on anterior acrosomal region only in caput and corpus spermatozoa, whereas in cauda and vd spermatozoa, it was found to be localized on anterior as well as post acrosomal region. In fixed-permeabilized cell (Figure 3), p14 was found to be localized only in anterior acrosomal region of caput. In corpus, cauda, vd spermatozoa on the other hand the protein was present in anterior as well as post-acrosomal region. FACS analysis showed the expression of p14 in 70–80% of live as well as fixed-permeabilized cells from all epididymal part (Figure 4).

### Presence of p14 in plasma membrane and soluble fraction of acrosome

**Figure 5:**
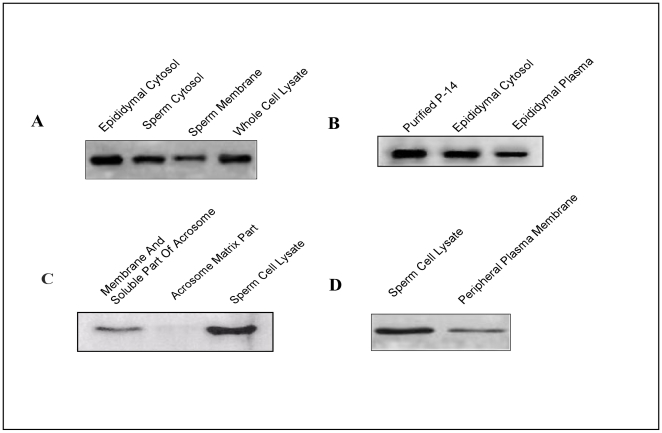
Presence of p14 in plasma membrane and soluble part of acrosome. (**A**) Western blot analysis showing the expression of p14 in membrane and cytosolic fractions of sperm and whole cell lysate. (**B**) Expression of the p14 in the cytosolic fraction of epididymis and cauda epididymal plasma. (**C**) Western blot showing the presence of p14 in plasma membranes and soluble components of the acrosome, acrosomal matrix and sperm cell lysate. (**D**) Immunoblot with a peripheral plasma membrane protein extract prepared from washed caudal epididymis following treatment with AES solution and cell lysate. Proteins were extracted from caudal sperm with 0.625% Triton-X-100 solution for immunoblotting (details in methods). Experiments were repeated four times.

As p14 was found to be present on sperm surface as well as intracellular region (above), we next sought to investigate its expression level in the different subcellular region. Western blot analysis revealed that although the protein is mostly cytosolic, it was found to be expressed in relatively low level in sperm membrane also (Figure 5A). Significant level of expression of the protein in epididymal plasma was also detected (Figure 5B). When plasma membranes and soluble components of acrosome were extracted from sperm with 0.625% Triton-X-100 solution for immunoblotting, low level of expression of p14 was observed in the soluble part but was absent in detergent insoluble matrix fraction (Figure 5C). Immunoblot with protein extracted from peripheral plasma membrane of washed caudal spermatozoa following treatment with 320 mM sucrose solution containing 1 mM EDTA and 1 mM ATP (AES) revealed low level of expression of this protein (Figure 5D).

### Effect of capacitation and acrosome reaction on the distribution of sperm p14

**Figure 6:**
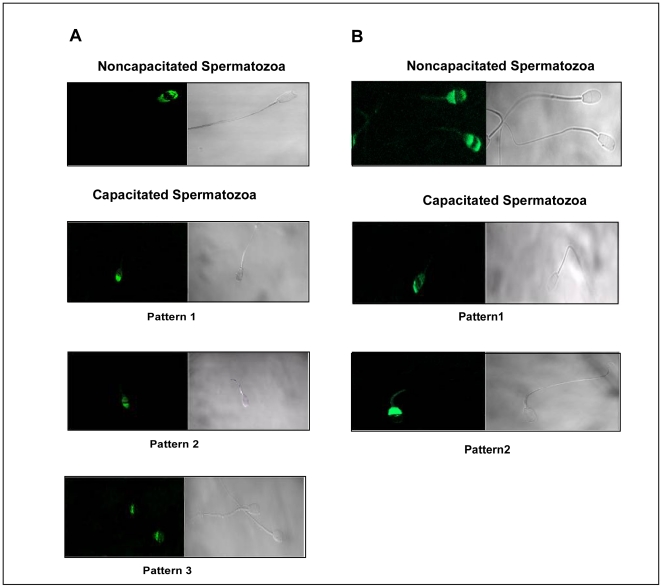
Effect of capacitation on the distribution of sperm p14. (**A**) Indirect immunofluorescence of anti-p14 labeled protein in live capacitated caprine spermatozoa. Upper panel showing the fluorescent and corresponding phase contrast micrographs of noncapacitated sperm (control). Lower panels showing the same in sperm capacitated for 3 hrs. Three different patterns of staining were observed. (**B**) Indirect immunofluorescence of anti-p14-labeled protein in fixed/permeabilized caprine sperm. Upper panels showing the fluorescence and corresponding phase contrast pattern of noncapacitated sperm. Lower panels correspond to the same but for 3 hrs capacitated spermatozoa. Two different patterns of immune-fluorescence were observed. The calculated percentages given as the mean ± SEM of at least four (n = 4) different experiments.

**Figure 7:**
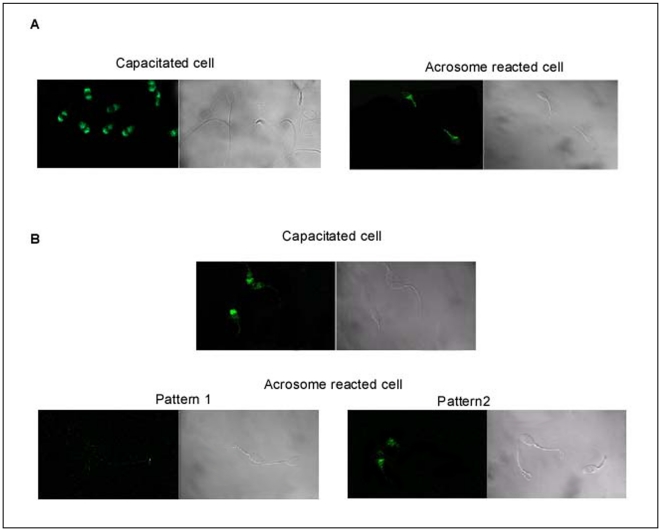
Distribution of p14 during sperm acrosome reaction. (**A**) Indirect immunofluorescence of anti-p14-labeled protein in acrosome reacted fixed caprine sperm permeabilized with paraformaldehyde. Left panel indicating the fluorescent and corresponding phase contrast photographs of sperm previously capacitated for 3 h but not acrosome reacted (control). Right panel showing the same in capacitated spermatozoa, later induced to acrosome react. (**B**) Indirect immunofluorescence of anti-p14-labeled protein in acrosome reacted fixed and nonpermeabilized caprine sperm. Upper panel showing the fluorescence and corresponding phase contrast pattern of sperm previously capacitated for 3 h but not acrosome reacted (control). Lower panels corresponding to the same but in capacitated spermatozoa, later induced to acrosome react. Two different patterns of immune-fluorescence were observed. The calculated percentages given as the mean ± SEM of at least four (n = 4) experiments.

Following determination of the expression status of p14, we next explored the changes in its distribution pattern during capacitation and acrosomal reaction. On incubating the spermatozoa under capacitating conditions, a change in the immunofluorescence pattern was observed. In non-permeabilized cells, three different patterns were found (Figure 6A). The majority of the cells showed a strong labeling in the anterior acrosomal region (66.2±5.4%, mean ± SEM) (n = 4). Significant number of cells (26.3±1.8%) were strongly labeled at anterior acrosomal region and faintly labeled in the post acrosomal region. Few cells (7.3±6.4%) showed faint labeling only in the post acrosomal head region. On the other hand, about 71.0%±6.8% (n = 4) of fixed/permeabilized cells showed intense labeling both in the anterior and post acrosomal head regions. 28.5±7.5% (n = 4) of the cells expressed p14 only in the post acrosomal region (Figure 6B). The pattern of distribution and number of cells showing this pattern did not change significantly with increase in capacitation time (data not shown). When capacitated cells were challenged with the calcium ionophore, A23187, to acrosome react, the pattern of labeling changed in the anterior acrosomal region of fixed/permeabilized sperm cells compared with cells incubated under capacitating conditions (Figure 7A). In nonpermeabilized spermatozoa, a weak signal (Figure 7B) was observed in 29.3±3.8%(n = 4) of cells. On the other hand, 69.7±5.8% of the cells showed almost no labeling in any region of spermatozoa cell.

### Expression level of p14 during capacitation and acrosome reaction at different time periods

**Figure 8:**
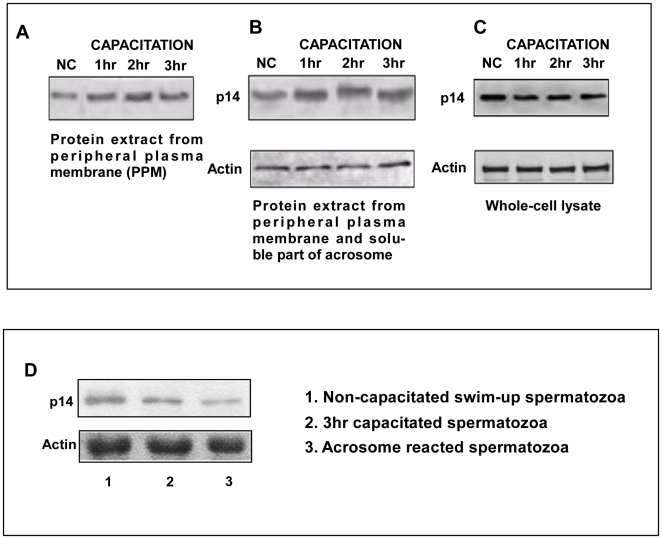
Expression level of p14 during different time period of capacitation and acrosome reaction. (**A**) Western blot analysis showing the expression of p14 in peripheral plasma membrane (PPM) of swim-up spermatozoa incubated under capacitated condition for different time periods. (**B**) Immunoblot for the presence of p14 in membranes and acrosome soluble component and (**C**). Whole cell lysate prepared from spermatozoa capacitated for 1, 2, and 3 hrs. (**D**). The expression of p14 in cell lysate of acrosome reacted spermatozoa previously capacitated for 3 hrs.

Next, to address how the distribution of p14 changes upon induction of capacitation, p14 level was checked in PPM, total membranes and soluble components of the acrosome, and whole cell lysate during capacitation. The result showed that during first hour of capacitation, p14 level increased in peripheral plasma membrane (Figure 8A) and possibly in acrosome (Figure 8B) but its level in whole cell lysate remained the same (Figure 8C) in non-capacitated and capacitated cells. Protein level in these membrane fractions and whole cell lysate did not change significantly with increase in capacitation time from 1 hr to 3 hrs. All these findings (Figure 6 and Figure 8) together confirmed that upon induction of capacitation, significant changes occur in the membrane expression level of p14. In acrosome reacted cells, p14 level was found to be decreased when compared with 3 hrs capacitated cells (Figure 8D).

### Effect of anti-p14 antibody on the forward motility retarding ability of p14

#### Microscopic Analysis

**Figure 9:**
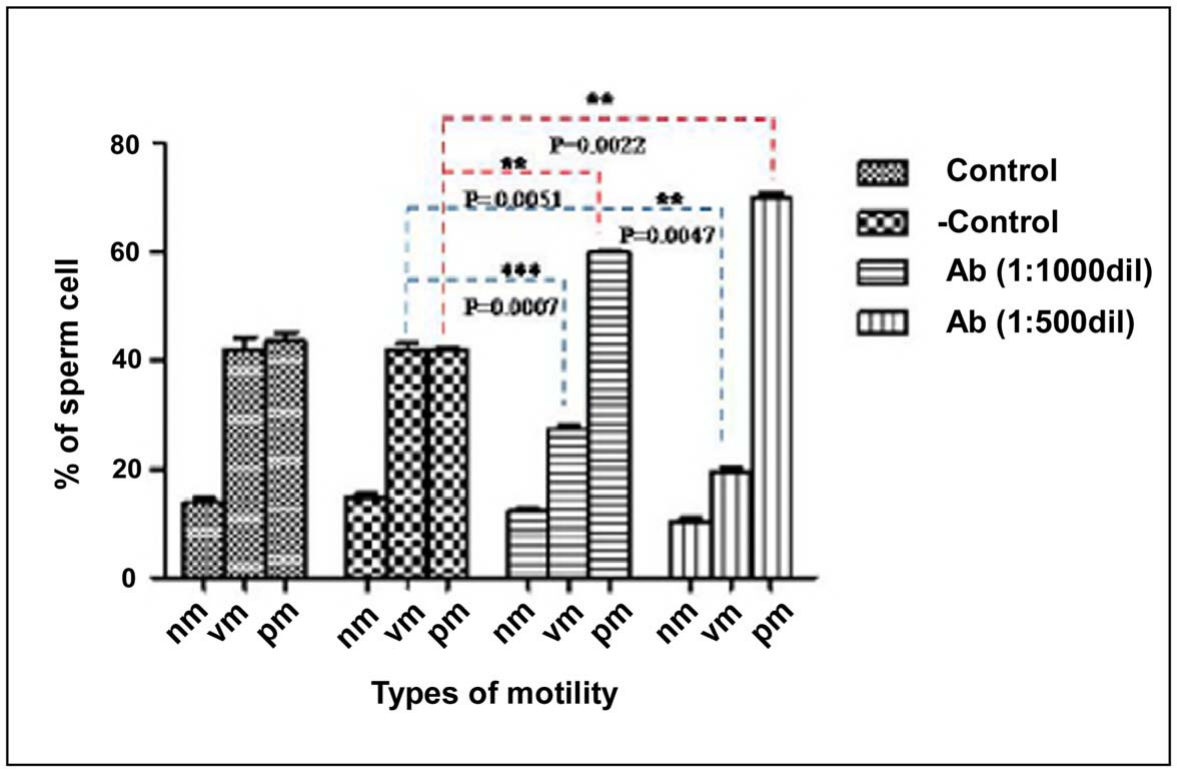
Effect of anti-p14 antibody on the forward motility retarding effect of p14: Microscopic analysis. Spermatozoa (1×10^6^) were incubated with anti-p14 antibody at different dilution (1∶500, 1∶1000) for 15 mins at room temperature (32°C±1) and forward motility of sperm was monitored. Data representing the mean of four experiments (n = 4) ± SEM. Units: nm = non-motile sperm, vm = vibrating sperm, pm = progressively motile sperm.

Previously it was reported from our laboratory that incubation of sperm cells with p14 inhibit forward motility of caprine spermatozoa in a concentration dependent manner [22], however its presence on sperm surface was not known. In the present study, sperm cells were incubated with anti-p14 antibody to block sperm surface p14 and monitored the number of progressively motile sperm cells with haemocytometer. The result showed that p14-antibody at a dilution of 1∶500 increased the number of progressively motile sperm cells from ∼42% (in control pre-immune serum treated cell) to ∼70% (Figure 9, Table 1). Low motile sperm populations were taken for microscopic assay to determine the motility promoting activity of p14 antibody.

**Table 1:** Microscopic analysis of sperm motility in the absence and presence of anti-p14 antibody.

Type of motility	Control cells	PI treated cells	Antibody treated cells	
	(Mean ± SEM)	(Mean ± SEM)	(Mean ± SEM)	
		1∶500 dilution	1∶1000 dilution	1∶500 dilution
Nm	14.167±1.041	14.67±1.528	12.37±0.635	10.4±1.002
Vm	42.3±3.559	42.10±1.852	27.5±1.323	19.68±1.473
Pm	43.5±2.838	41.90±1.153	59.97±1.31	70.03±1.732

Nm = Nonmotile sperm, Vm = Vibrating sperm, Pm = progressively motile sperm, PI = preimmune sera. Low motile sperm populations were taken for microscopic analysis to determine the motility promoting activity of p14 antibody.

#### Analysis by CASA

**Figure 10:**
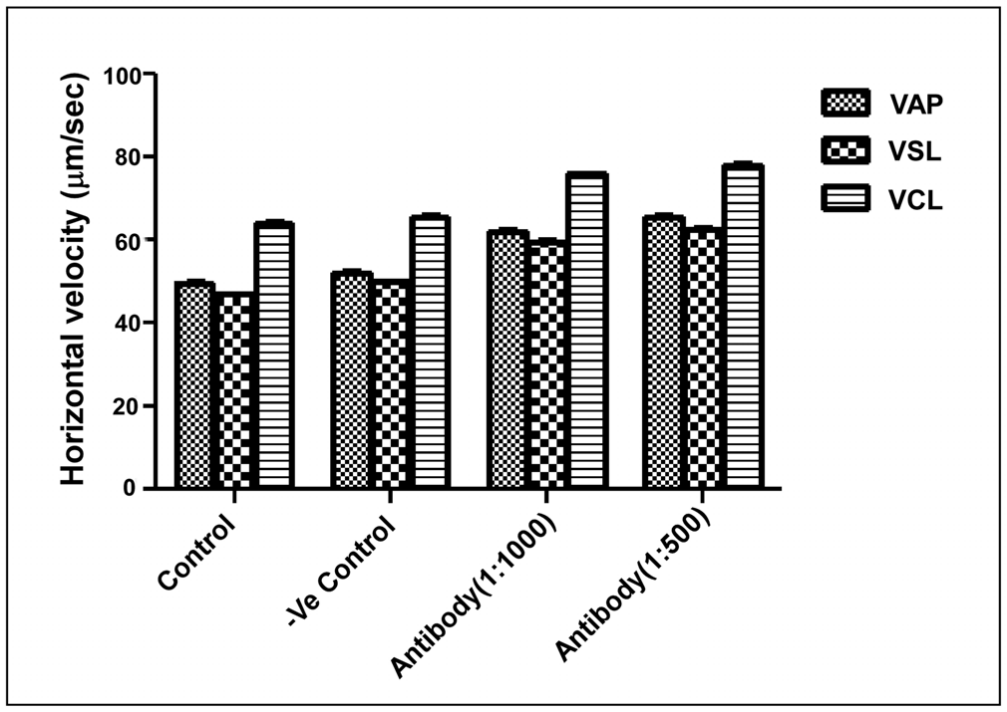
Effect of anti-p14 antibody on the forward motility retarding effect of p14: CASA. Data showing the effect of anti-p14 antibody on different CASA parameters. Data represents mean of three experiments (n = 3) ± SEM. Units: VAP (Average Path Velocity) = µm/sec, VSL (Straight Line Velocity) = µm/sec, VCL (Curvilinear Velocity) = µm/sec.

Addition of 1∶500 dilution of anti-p14 antibody to the sperm preparation caused significant increase in sperm forward motility. Two different doses, 1∶500 and 1∶1000 dilutions of anti-p14 antibody were used to see the effect on motility variables of CASA. There was approx. 26% increase in VAP, 26% in VSL and 19.4% in VCL at higher concentration of the antibody (Figure 10, Table 2).

**Table 2:** Sperm motility study by CASA in the absence and presence of anti-p14 antibody.

Treatments	VAP	VSL	VCL
	(Mean ± SEM)	(Mean ± SEM)	(Mean ± SEM)
Control	49.775±0.376	46.925±0.290	64.233±0.203
-ve Control	52.167±0.433	49.867±0.318	65.500±0.651
Antibody(1∶1000)	62.225±0.189	59.467±0.593	75.833±0.088
Antibody(1∶500)	65.608±0.231	62.683±0.277	78.175±0.275

Here control sample is only sperm cell in Ca^2+^ free Ringer solution, and –ve control is the pre-immune sera treated cells in the same medium. Assays were performed using sperm collected from at least six different tissue samples. VAP (Average Path Velocity) = µm/sec, VSL (Straight Line Velocity) = µm/sec, VCL (Curvilinear Velocity) = µm/sec.

### Effect of anti-p14 antibody on ionophore induced acrosome reaction

**Figure 11:**
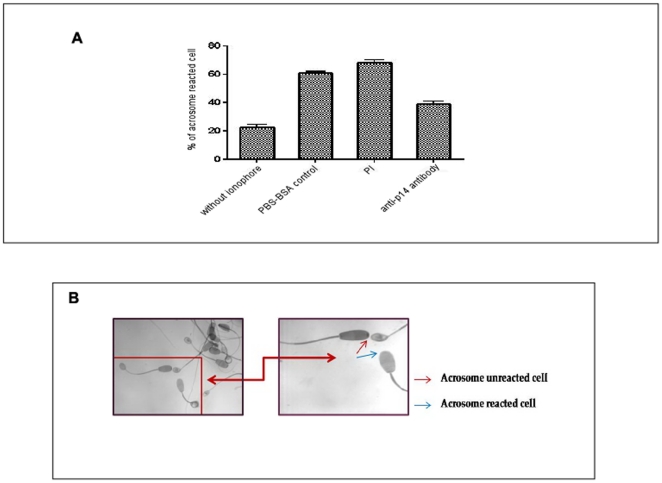
Effect of anti-p14 antibody on acrosome reaction of cauda spermatozoa monitored by Rose Bengal staining method. Acrosome reaction was carried out under the standard assay conditions and the cells after staining with Rose Bengal were observed under microscope at 1,000× magnification. Sperm cells treated with anti-p14 antibody. (**A**) Data showing the percent of acrosome reacted cells at different assay conditions (without ionophore, and PBS–BSAcontrol, Pre-immune sera, anti-p14 antibody in presence of ionophore). (**B**) Red arrow represents acrosome unreacted (acrosome intact) and blue arrow represents acrosome reacted (acrosome not intact) sperm. The “acrosome unreacted” cell has a well defined tiny colored spot on the tip of the sperm head whereas “acrosome reacted” cell has no such colored spot.

Effect of anti-p14 antibody on sperm acrosome reaction was studied by Rose Bengal staining method. Incubation of sperm cells with anti-p14 antibody, before induction of acrosome reaction, decreased the number of acrosome reacted cells from 68.0±3.6% in negative control to 38.7±3.5% in antibody treated sample (Figure 11A, Table 3). Acrosome intact or unreacted cells were identified by a red colored spot on the tip of sperm head. Acrosome reacted cells did not show any such spot (Figure 11B). Acrosome reaction was also assessed by chlortetracycline assay and similar result was obtained (data not shown). The calculated percentages are given as the mean ± SD of at least four experiments.

**Table 3:** Effect of anti-p14 antibody on caprine sperm acrosome reaction.

Treatments	Acrosome reacted sperm (%), mean ± SD
PBS – BSA control	60.66±2.52
Preimmune sera (1∶1000 dil)	68.00±3.6
P14 antibody (1∶1000 dil)	38.67±3.5

Acrosome reaction was induced by calcium ionophore A23187. Assays were performed using sperm collected from at least four different testis samples. Control values in absence of ionophore were 22.66±3.05.

## Discussion

Mammalian sperm-egg interactions are mediated by specific complementarity of proteins, glycoproteins, and carbohydrates present at the surface of gametes [25], [26]. Before spermatozoa can interact with the zona pellucida, which is an extracellular matrix surrounding the egg, they are subjected to post-testicular modifications yielding male gametes that exhibit forward motility and oocyte-binding ability [27]. Epididymal secretory products are involved in the acquisition of fertilizing ability by mammalian spermatozoa. Although many epididymal proteins have been described, the function of the majority of them in sperm physiology remains to be clarified. After epididymal transit, male gametes undergo other complex surface transformations that occur when they encounter accessory gland secretions during ejaculation as well as within the female genital tract [28]. Physiological modifications of the spermatozoa within the female reproductive tract have been extensively studied and collectively defined as capacitation [29]. Capacitation represents the completion of sperm maturation that confers on mammalian sperm the acquisition of fertilization competence either in vivo or in vitro. As spermatozoa progress through capacitation, dramatic changes occur in the sperm membrane and in surface protein distribution [30], [31]. In vivo, sperm egg interaction triggers Ca**^2+^** influx in sperm, which in turn activates a series of biochemical events leading to the phenomenon of membrane fusion [32]. It can be induced in vitro in capacitated spermatozoa by incubation with solubilized zona pellucida, progesterone, epidermal growth factor, or by Ca**^2+^**/2H^+^/ionophore A23187 [33].

Recently we have reported the isolation and characterization of a14 kDa protein (p14) from goat epididymal cytosol [12]. In the present work the localization of p14 in spermatozoa has been described. It has been observed that in total epididymal tissue, p14 level is increased gradually while in individual sperm cell, the level remains the same from caput to vd. This clearly indicates that p14 level specifically rises in epididymal epithelial tissue during epididymal transition of spermatozoa. Immunohistochemical study confirms its presence in luminal sperm cells and epithelial cells of epididymis. Indirect immunofluorescence of non-permeabilized spermatozoa reveals the localization of the protein at the anterior acrosomal region of the head in caput and corpus spermatozoa, while in cauda and vd spermatozoa, it is observed in anterior and post acrosomal region. This suggests that p14 is expressed at the sperm surface, facing the extracellular milieu. A strong signal, however, has been detected on the anterior region of the head of fixed-permeabilized caput spermatozoa, on the anterior as well as post acrosomal region of corpus, cauda and vd spermatozoa. Our findings thus strongly supports that a population of p14 is also expressed intracellularly which is also evident from the FACS analysis. Subcellular localization experiments reveal that the protein is mainly cytosolic, although a significant level of expression is also observed in membrane. The detail physiological significance of why p14 is located more or less in all compartments of the sperm head needs further investigation.

As the interior of the acrosome is compartmentalized biochemically and morphologically, a given protein of the acrosomal lumen may be considered as a soluble constituent or an acrosomal matrix component depending upon whether the protein is solubilized following extraction with Triton X-100 under conditions that block proteolysis. Now in our study when membranes and soluble components of acrosome were extracted from sperm with 0.625% Triton X-100 solution for immunoblotting, results indicate that the protein is present in the detergent extract with complete absence in the insoluble matrix fraction. This suggests that along with cytosol, the protein resides in the membrane and/or the soluble fraction of acrosome but not in the acrosomal matrix. Western blot analysis of protein extracted from peripheral plasma membrane with AES solution confirms its presence in this membrane fraction. During capacitation, p14 level is found to be increased in peripheral plasma membrane and probably in acrosome also. However, its level in whole cell lysate remains the same indicating no new synthesis or breakdown of p14 during this process. Indirect immunofluorescence with live cells confirms modification in the surface distribution pattern of p14 during sperm capacitation. Majority of the capacitated cells (66%) show strong labeling only on the anterior acrosomal region. Other cells show either strong labeling in anterior acrosomal region and faint labeling in post-acrosomal region (26%) or faint labeling only in post-acrosomal region. The pattern does not change significantly with time. In the fixed-permeabilized capacitated cells, a decrease in the signal intensity is observed in the anterior acrosomal region of few cells but majority of cells show labeling in anterior as well as post- acrosomal region. All these findings together strongly suggest the change of distribution of p14 upon induction of capacitation thereby confirming the involvement of this protein in the process of caprine sperm capacitation. On the other hand, when capacitated spermatozoa is challenged to acrosome react, the signal is found to be decreased significantly in most permeablized and non-permeabilized cells indicating loss of the protein from sperm plasma membrane/outer-acrosomal membrane. Thus, all our findings cumulatively suggest that p14 is mainly cytosolic, also associated with the outer acrosomal membrane, or associated with the plasma membrane, but not with the inner acrosomal membrane. Capacitation reaction induces a change in the distribution of the protein from post acrosomal to anterior acrosomal surface. During acrosome reaction p14 is lost from membranes over the anterior head region which again confirms that the protein is not present on inner acrosomal membrane (IAM).

One of the major reasons of male infertility is due to low/no sperm motility (asthenospermia) [13], [34]. Previously, it has been reported from our laboratory that incubation of sperm cells with p14 inhibits forward motility of caprine spermatozoa in a concentration dependent manner [22]. In the present study anti-p14 antibody has been used to block sperm surface p14. The results from CASA and microscopic analysis show that blocking sperm surface p14 enhances the progressive motility of sperm cells. Although no clear mechanism in support of this event is known till now, a change in serine-threonine phosphorylation of some spermatozoal proteins which might be associated with sperm motility could be responsible for this (results not shown). Treatment of sperm cells with anti-p14 antibody, before induction of acrosome reaction with Ca**^2+^**-ionophore A23187 decreases the number of acrosome reacted cells from ∼68% in negative control to ∼39% in antibody treated sample. Therefore it is suggested that p14 is probably involved in the acrosomal membrane fusion event. Blocking p14 by anti-p14 antibody may somehow hinders this fusion, thus decreases the number of acrosome reacted cells. However further investigations are required to delineate the exact biochemical mechanism in detail.
